# The Effect of Oil Additives on the Properties of Fly Ash-Based Foamed Geopolymers

**DOI:** 10.3390/ma17235819

**Published:** 2024-11-27

**Authors:** Barbara Kozub, Jan Dudek, Mykola Melnychuk

**Affiliations:** 1 Department of Materials Engineering, Faculty of Materials Engineering and Physics, Cracow University of Technology, 37 Jana Pawła II Street, 31-864 Cracow, Poland; jan.dudek@student.pk.edu.pl; 2 Department of Materials Science, Lutsk National Technical University, Lvivska 75, 43018 Lutsk, Ukraine; m.melnychuk@lntu.edu.ua

**Keywords:** foamed geopolymer, fly ash, vegetable oil, engine oil, porosity, mechanical properties, thermal conductivity, leachability

## Abstract

Geopolymers are a modern class of construction materials that show significant potential for sustainable development, especially through the use of industrial wastes such as fly ash. This study investigated the effect of different oil additives on the properties of fly ash-based geopolymers, with particular emphasis on the use of both new and used oils. Test samples were prepared using class F fly ash and a 10-molar solution of sodium hydroxide and an aqueous solution of sodium silicate. Oil additives were added at 5%, 10% and 15% by weight. The physical and mechanical properties of the samples were assessed by measuring density, thermal conductivity, compressive and flexural strength, and by analyzing porosity and microstructure. The results showed that oil additives significantly affected the pore structure and mechanical properties of the geopolymers. Furthermore, the type and condition of the used oil determined the mechanical properties, including compressive and flexural strength. Research indicates the possibility of using oils as additives to geopolymers, which helps improve their physical properties and promotes sustainable development through recycling oil waste.

## 1. Introduction

Geopolymers are a class of inorganic polymers derived from aluminosilicate materials, including fly ash, granulated blast furnace slag, and metakaolin. Geopolymers are gaining increasing popularity due to their potential use in various areas, such as the construction sector, as a sustainable alternative to traditional cementitious materials, in the chemical industry, and in the remediation of contaminated water and soil. Their formation results from the dissolution of aluminosilicate precursors in an alkaline solution, which leads to the formation of a three-dimensional network structure. This structure gives geopolymers their characteristic mechanical and chemical properties, including high strength and resistance to aggressive environmental factors [1,2,3,4]. Compared to traditional building materials such as Portland cement, geopolymers have a smaller carbon footprint, making them an attractive alternative from a sustainability perspective [5].

By carefully selecting raw materials and synthesis conditions, one can tailor the properties of geopolymers to suit their potential applications [6,7,8,9]. Foamed geopolymers, which combine the advantages of traditional geopolymers with lightness and thermal insulation properties, have significantly advanced the field of construction materials. Foaming agents such as hydrogen peroxide or aluminum powder, when incorporated into geopolymers, create a porous structure without significantly altering the chemical structure of their matrix. This makes them more thermally insulating while decreasing their density [10,11,12,13]. Moreover, due to their inorganic nature, foamed geopolymers are characterized by high fire resistance, which further increases their potential for use in building materials [14,15,16,17]. In contrast, the synthesis of foamed geopolymers from industrial by-products such as fly ash and metakaolin is in line with sustainable construction practices, promoting the use of environmentally friendly alternatives in the construction industry [18,19].

Similarly to the flotation separation of minerals, where biodegradable Tamarindus indica polysaccharide enables selective calcite depression while maintaining fluorapatite buoyancy [20], the introduction of calcium lignosulfonate improves the efficiency of brucite and serpentine separation by reducing the transport of fine-grained serpentine to the foam [21]. Additionally, the modification of geopolymers by organic additives, such as oils, affects the surface and separation properties [22]. The use of oils as additives in geopolymers leads to the improvement of the porous structure and mechanical properties by regulating adhesion and hydrophobicity; this may resemble the selective interaction of biodegradable Tamarindus indica polysaccharide with selective calcite while maintaining fluorapatite,. Thanks to this improvement, both flotation and geopolymerization, through precisely selected modifiers, allow for obtaining the expected separation and strength properties of materials while maintaining a sustainable approach to the environment.

The incorporation of additives such as oils into foamed geopolymers is a promising research direction, as it can significantly affect their porosity, mechanical strength, and thermal insulation properties. Saponification of oils in the alkaline environment of the geopolymer matrix leads to the formation of macropores, which improve the mechanical and thermal insulation properties of the material [18,23,24,25].

There are several ways to introduce oils into cement or geopolymer paste. Three methods are described in the literature: direct introduction of oil, the pre-emulsion process and the solid impregnation process [22].

The direct introduction of oil additives, used in this work, is a popular method due to its simplicity and the possibility of mixing all the elements in one container, which is called “mixing in one vessel” [26]. Active suspensions take part in the process of creating cement materials, and the addition of a water-immiscible liquid resembles the creation of an emulsion, where the dispersed liquid tends to coalesce spontaneously [22,27]. The main advantage of this method is the high viscosity of the cement suspension, which increases shear stresses, favoring the formation of smaller pores [28].

The development of the pre-emulsification method aimed to mitigate the risk of phase separation between cement and oil. In this case, the oil is pre-emulsified in the activating solution before the addition of solid precursors [22,29]. In this case, however, the use of stabilizers such as surfactants is required to maintain emulsion stability and control droplet distribution [30].

Impregnation, a third method, involves adsorbing the organic liquid onto the solid material before introducing it into the cement paste. This eliminates the risk of phase separation, and the lack of a need for surfactants simplifies the process. This method is more stable and has less impact on the mechanical properties of the final composite, even though it only allows the introduction of small amounts of liquid [22].

In addition to improving porosity and thermal properties, adding oil to foamed geopolymers is associated with improved oil absorption capacities. Silveira Maranhão et al. [31] in their study showed that oil-enriched geopolymers can achieve high oil removal efficiency. The oil retention capacity of foamed geopolymers is due to their porous structure, which creates a large surface area for absorption. This makes them promising materials for applications in environmental remediation, especially in the case of oil spill cleanup [31,32].

This paper aimed to investigate the effect of different oil additives on the properties of fly ash-based foamed geopolymers, with emphasis on the aspect of using both new and post-use oils. This approach not only allows for examining the effect of applied modifications on the properties of materials, but also fits into the idea of sustainable development through the recycling of waste oils.

This study investigates the effects of both new and used oils (vegetable and engine oils) on the properties of fly ash-based geopolymers and, consequently, the potential for using industrial waste products in geopolymer matrices. Existing studies have focused mainly on single types of new oils or chemical additives, not considering the effects of waste oils that contain oxidation by-products and other contaminants. The conducted research aims to expand our knowledge on the effect of oils on pore formation processes and the mechanical and physical properties of foamed geopolymers based on fly ash.

## 2. Materials and Sample Preparation

The test samples were prepared based on class F fly ash from the Skawina CHP Plant (Skawina, Poland). In order to achieve the assumed research objective, vegetable and engine oil were used as additives, both in the delivery state and after use, in quantities of 5, 10 and 15% by weight in relation to the fly ash mass.

A 10-molar solution of alkaline sodium hydroxide (NaOH) and an aqueous solution of sodium silicate R-145 (with a molar module of 2.5 and a density of about 1.45 g/cm^3^) were mixed in a volume ratio of 1:2.5 for the geopolymerization process. The foaming agent in the geopolymer production process was 36% hydrogen peroxide (Grupa Azoty, Puławy, Poland), which was added in the amount of 30 mL to each of the mixtures. As additives to stabilize the foaming process, 10% by weight of gypsum plaster (Dolina Nidy, Leszcze, Poland) and 5% by weight of hydroxyethylcellulose (Glen-tham Life Sciences, Corsham, UK) were used.

The mixtures were prepared using a planetary mixer. The first step was to mix the dry ingredients, fly ash, and stabilizing agents; mixing was carried out for 4 min. Next, an alkaline solution was introduced into the vessel, ensuring a liquid-to-solid ratio of 0.4. The mixing was continued until a lump-free, uniform, and smooth structure of the geopolymer paste was achieved (the mixing time was about 6 min). In the next step, oil additives were added to the geopolymer paste prepared this way, and mixing was continued for another 4 min until the oils were evenly distributed in the paste. Then, the foaming process was started. The foaming process was carried out by adding 30 mL of 36% hydrogen peroxide as a foaming agent to each of the prepared mixtures. The paste was mixed for about 2 more minutes after adding the foaming agent. The prepared geopolymer paste was poured into previously prepared molds. The prepared samples were cured in a laboratory furnace (POL-EKO-APARATURA, Wodzisław Śląski, Poland) at 75 °C for 24 h.

Table 1 shows the symbols used for the prepared samples; they are based on the amount of oil introduced and its type.

## 3. Research Methodology

### 3.1. Fly Ash Particle Size Measurement

The analysis of the fly ash particle size distribution was performed using a laser particle analyzer from Anton Paar GmBH (Graz, Austria), which uses the light diffraction technique. The measurement was performed in wet mode, which required the preparation of a sample of ash and its introduction into the device, where the light scattering analysis was then performed. The study determined the statistical values of grain size based on three measurements.

### 3.2. Fly Ash XRD Analysis

The phase composition of the fly ash samples was investigated using X-ray diffraction (XRD) analysis. The study was performed on a PANalytical Aeris X-ray diffractometer (PANalytical B.V., Almelo, The Netherlands). Rietveld phase analysis was performed using the PDF-4+ database provided by the International Center for Diffraction Data (ICDD).

### 3.3. Fly Ash XRF Analysis

The study used the XRF fluorescence X-ray spectrometry method to identify and determine the oxide composition of the fly ash. The study utilized the EDX-7200 spectrometer from SHIMATZU (SHIMADZU Europa GmbH, Duisburg, Germany).

### 3.4. Density and Thermal Conductivity Measurement

The density of the prepared geopolymer samples was determined using the geometric method. Based on the measured dimensions of the samples and their mass, the volume was determined, and then the density. The measurements were carried out on 4 samples, based on which the standard deviation was determined.

The thermal conductivity coefficient was measured using a lambda meter HFM 446 Lambda Series by NETZSCH (Netzsch GmbH & Co., Selb, Germany). The measurement was carried out on samples with dimensions of 190 mm × 190 mm and height of 40 mm, without the use of thermocouples, in the temperature range from 0 to 20 °C.

### 3.5. Strength Tests

Samples for bending strength tests were cut from plates intended for thermal conductivity measurements. The dimensions of the samples were 40 mm × 40 mm × 180 mm. The three-point bending test was performed on a universal testing machine Auto-graph AGS-X by SHIMADZU (Shimadzu, Kyoto, Japan), at a speed of 5 mm/min, with a support spacing of 150 mm, in accordance with PN-EN 12390-5:2019-08 [33]. The bending strength measurement was performed on four samples for each of the tested mixtures.

Compressive strength was measured on halves of the samples after bending tests in accordance with PN-EN12390-3:2019-07 [34], at a speed of 0.05 MPa/s, using a Matest testing machine with a force of 3000 kN (Matest, Treviolo, Italy). The compressive strength measurement was performed on eight samples for each of the tested mixtures.

### 3.6. Measurement of Porosity

The porosity of the samples was assessed based on the images of the sample cross-sections taken on a Keyence VHX-E100 digital microscope (KEYENCE INTERNATIO-NAL, Mechelen, Belgium). For the calculation purposes, images of the cross-sectional area, perpendicular to the direction of pouring the geopolymer paste into the molds, were taken of the plates previously used for thermal conductivity measurements. Measurements were taken on a series of 10 images from the sample cross-section. The images were analyzed using ImageJ 1.54g software (https://imagej.net/ij/, accessed on 30 October 2024), determining the average pore size and porosity fraction. The micrographs were previously appropriately prepared by cropping, converting to grayscale, and applying the threshold filter. The default settings for the threshold filter were selected. The regions from 0 to 128 in the grayscale were chosen for analysis; pixels in the image are highlighted in red for calculations.

### 3.7. Structure Observation

The microstructure observations of the studied geopolymers were performed using a JEOL JSM-820 scanning electron microscope (IXR Inc., Austin, TX, USA). Before observation, the samples were prepared by sputtering with a gold layer in a JOEL DII-29030SCTR vacuum coater (IXR Inc., Austin, TX, USA), which enabled obtaining high-quality images of the geopolymers’ microstructure.

### 3.8. Leachability Studies

Five types of samples, including the reference sample 100FA and samples with 15% vegetable and engine oils (new and used), underwent leachability tests. In order to collect material for testing, the samples were crushed and prepared for testing in accordance with the PN-EN 932-2:2001 [35] standard.

## 4. Results and Discussion

This chapter presents the results of tests of the physicochemical and mechanical properties of foamed geopolymers modified with vegetable and engine oils. The influence of the type and amount of added additives on the density, mechanical strength, thermal conductivity, porosity of samples, leachability, and structure is discussed.

### 4.1. Fly Ash Characteristics

**Figure 1:**
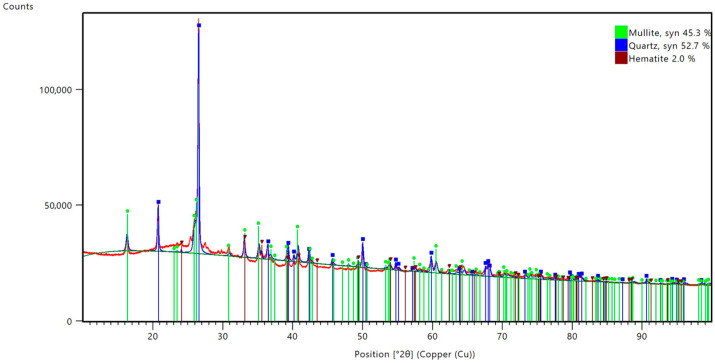
Diffractogram of the fly ash used in the study.

**Figure 2:**
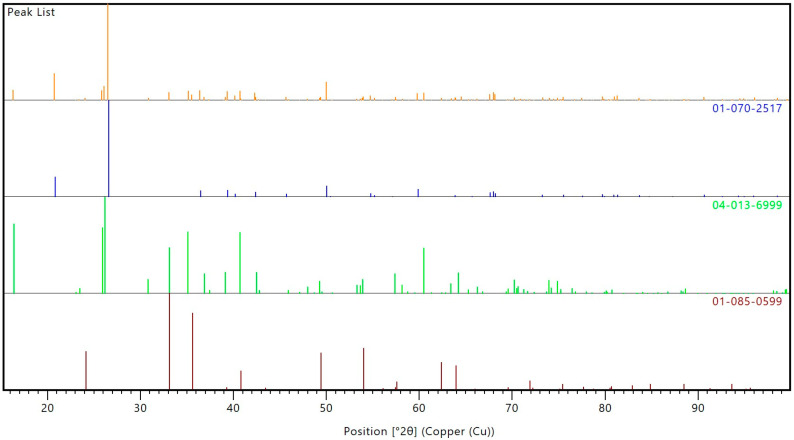
The peak list from XRD analysis.

The results of the phase composition analysis are presented in Figure 1. Figure 2 shows the peak list from the analysis. Based on the Rietveld phase analysis, it was determined that the fly ash used in the study mainly consisted of mullite (45.3%), quartz (52.7%) and hematite (2.0%).

**Figure 3:**
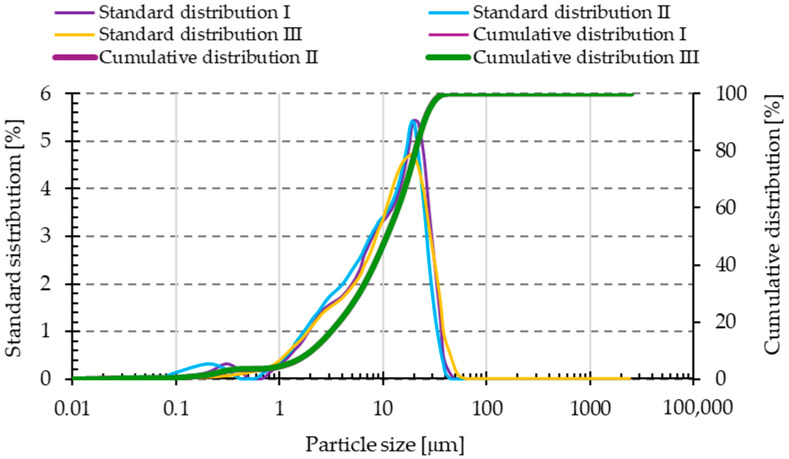
The particle size distribution and cumulative curves for fly ash.

Figure 3 shows the results of the analysis of the fly ash particle size distribution. Table 2 shows the statistical data from the analysis of the fly ash particle size distribution, including the values of the D10, D50 and D90 diameters, the average fly ash particle size and the span value.

The studies on the particle size distribution of fly ash showed that most of the particles are pretty small and spread out evenly, which is good for the geopolymerization process. The average particle size values were D_10_ = 2.06 μm, D_50_ = 10.97 μm and D_90_ = 25.06 μm, respectively. The fine particle fraction promotes better dissolution and reaction with alkaline solutions, which can lead to more uniform and stable geopolymer materials. In addition, the small size of the ash particles results in a larger reaction surface, which promotes an efficient process [36,37].

The analysis carried out using X-ray fluorescence spectrometry (XRF) allowed the determination of the oxide composition of the fly ash used for the research (Table 3), which mainly consists of silicon oxides (SiO_2_) and aluminum (Al_2_O_3_).

The obtained results of the XRD phase composition and XRF oxide composition analysis indicate that fly ash can be successfully used as a precursor for geopolymerization. It is characterized by an appropriate content of SiO_2_ and Al_2_O_3_ oxides, and their molar ratio is consistent with the literature indications [27,38].

### 4.2. Density

**Figure 4:**
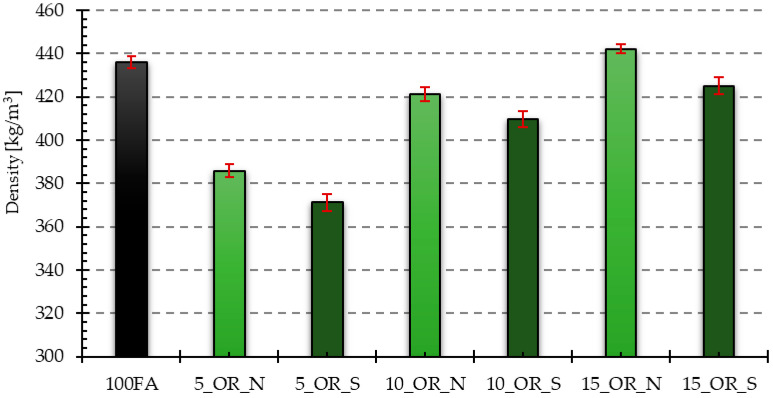
The density of the analyzed samples with vegetable oil added in the delivery state and after use, in relation to the reference sample.

**Figure 5:**
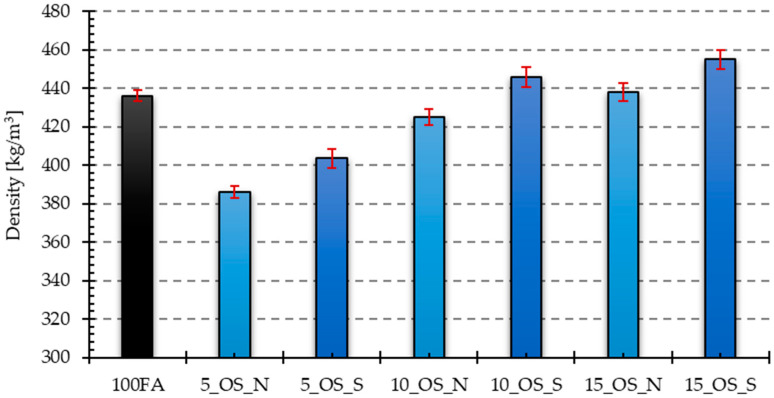
The density of the analyzed samples with engine oil added in the delivery state and after use, in relation to the reference sample.

Based on the measured dimensions of the samples and their mass, the volume and the density were determined. For each of the prepared mixtures, measurements were carried out on four samples, based on which the standard deviation was determined. Figure 4 and Figure 5 graphically present the obtained results from density measurements for all samples with reference to the reference sample 100FA, respectively, for the samples modified with the addition of vegetable oil and engine oil, both in the delivery state and after use.

As can be seen from the data presented above, in the case of samples with the addition of vegetable oil, the lowest density is characteristic of the sample with the addition of 5% oil after use, which is about 85% in relation to the reference sample, the density of which was 436.11 kg/m^3^. On the other hand, the highest density is characteristic of the sample with the addition of 15% new vegetable oil, which is about 1.4% higher in comparison to the reference sample. The analysis of the obtained results reveals that the introduction of the vegetable oil additive generally reduces the density of geopolymer samples, except for the previously mentioned sample that added 15% new vegetable oil. Furthermore, it can be noticed that in the case of using the additive of oil after use, the obtained density values are lower than in the case of using new oil introduced to the mixture in identical proportions.

In the case of samples with the addition of engine oil, the lowest density is characteristic of the sample with the addition of 5% new oil, which is about 88% in relation to the reference sample. The sample with 15% engine oil added after use has the highest density, approximately 4% higher than the reference sample. In the case of samples with engine oil added, certain dependencies can also be observed, i.e., with the increase in the percentage of oil in the mixture, an increase in their density is noticeable, and only for samples with 10 and 15% of oil added after use, these values were higher compared to the reference sample. Analyzing the effect of the oil condition, a different trend is observed in the case of samples with engine oil added than in the case of samples with vegetable oil. As a result, for the same weight shares of oil in the mixture, the obtained density values are higher for the variant using used oil.

### 4.3. Porosity

**Figure 6:**
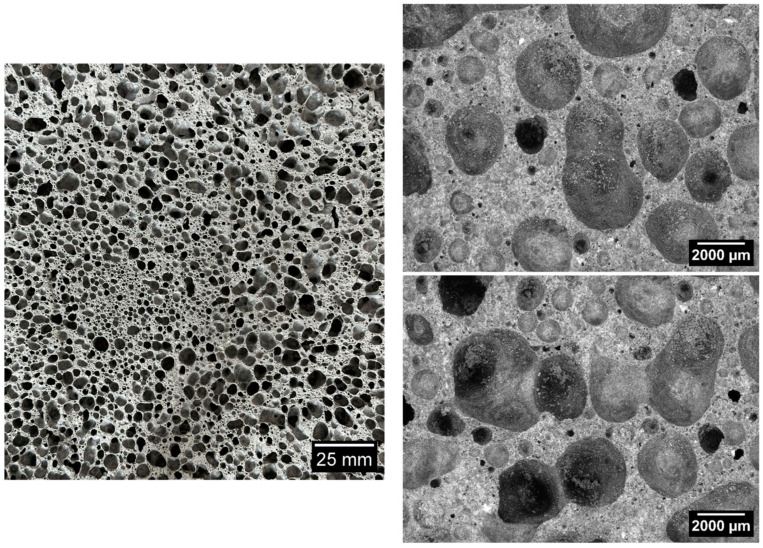
Micrographs of porosity of the sample marked as 100 FA.

**Figure 7:**
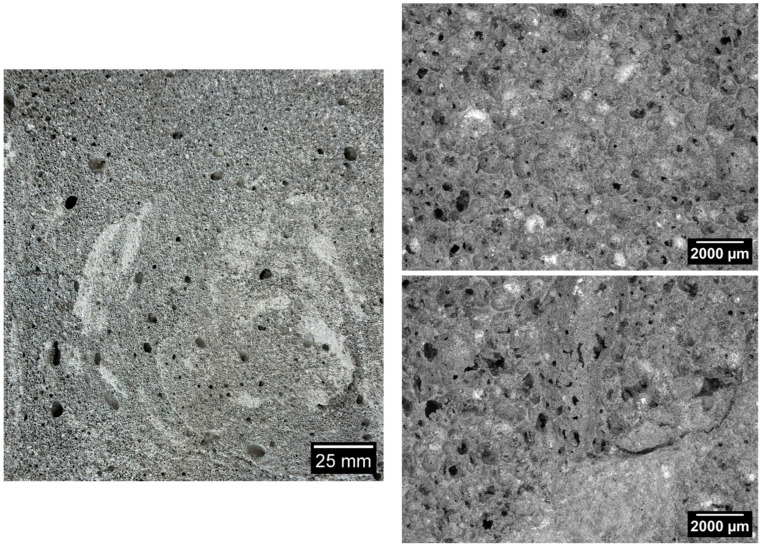
Micrographs of porosity of the sample marked as 15_OR_N.

**Figure 8:**
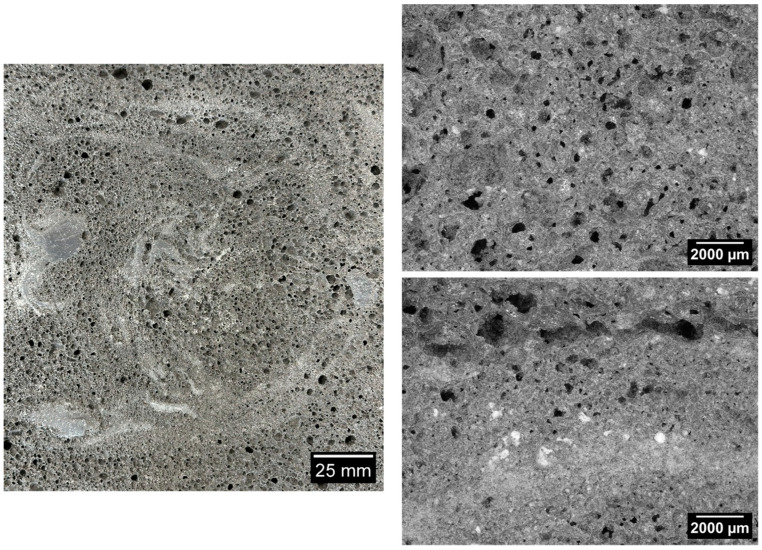
Micrographs of porosity of the sample marked as 15_OR_S.

**Figure 9:**
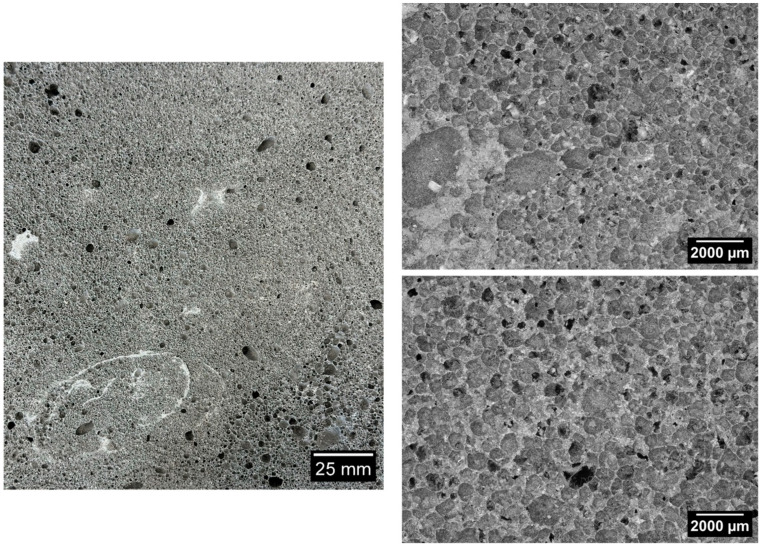
Micrographs of porosity of the sample marked as 15_OS_N.

**Figure 10:**
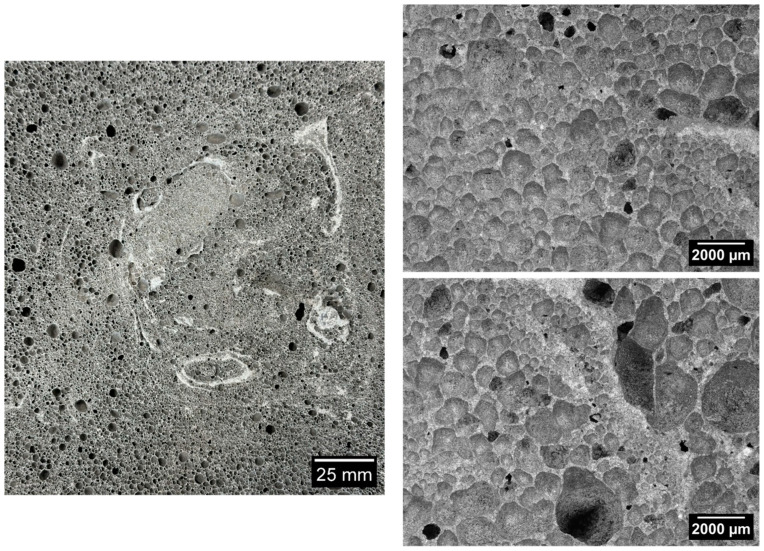
Micrographs of porosity of the sample marked as 15_OS_S.

Figure 6, Figure 7, Figure 8, Figure 9 and Figure 10 below show examples of photo combinations used to calculate the porosity share, average pore size and pore shape factors for the tested polymers.

Analyzing the porosity images taken for all the produced foamed geopolymers, it was possible to observe that the reference sample made on the basis of fly ash, without the addition of oil, was characterized by pores of the largest size, the most irregular shapes and their uneven dispersion. The introduction of vegetable and engine oils, both during delivery and after exploitation, resulted in a significant reduction in the size of pores and a more spherical shape compared to the reference sample. However, the cross-section of the samples with oil additives revealed areas of non-foamed material, whereas the addition of vegetable oil, both new and after exploitation, increased the proportion of these areas. Such areas did not occur in the case of the reference sample without the addition of oils. This may be related to the workability of the geopolymer paste; during the production of the samples, a decrease in the workability of the geopolymer paste was observed with the increase in the share of oils introduced into the mixture, which translated into an increase in the difficulty of casting the geopolymer paste.

**Figure 11:**
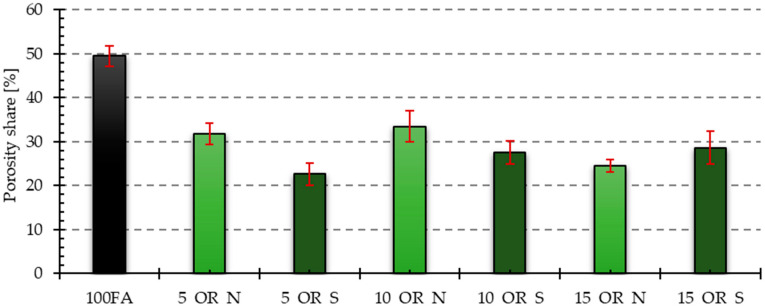
Results of porosity share for samples with vegetable oil addition.

**Figure 12:**
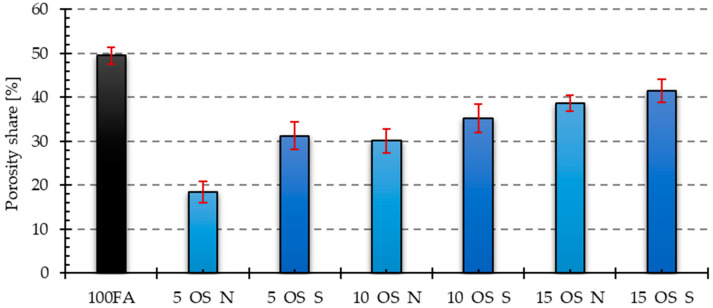
Results of porosity share for samples with engine oil addition.

**Figure 13:**
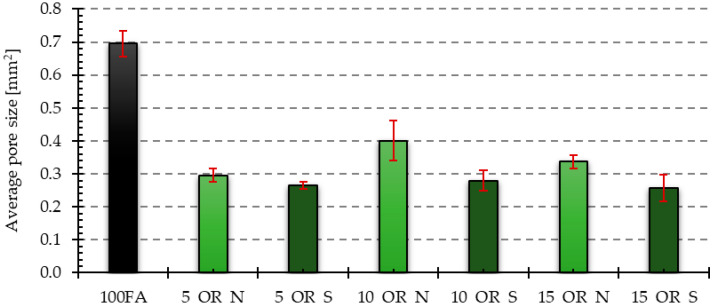
Average pore size results for samples with added vegetable oil.

**Figure 14:**
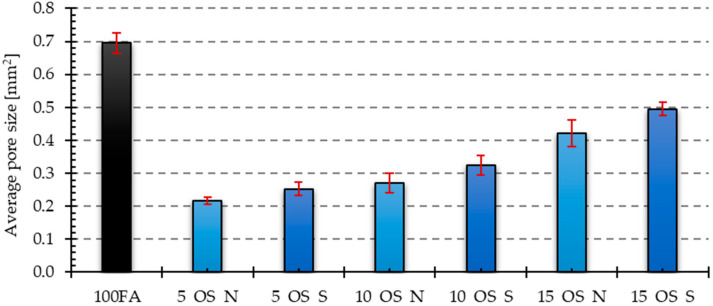
Average pore size results for samples with added engine oil.

The visual observations were confirmed by the results of the porosity analysis conducted in the ImageJ program. The results of the porosity share on the analyzed surface and the average pore size are presented in Figure 11, Figure 12, Figure 13 and Figure 14.

Both the samples containing vegetable oil and engine oil exhibited a lower porosity share in the tested cross-section compared to the reference sample. The sample containing 5% of old oil had the lowest porosity share among the samples containing vegetable oil. The condition of the oil also affected the porosity share, resulting in lower porosity in samples containing 5 and 10% of used vegetable oil compared to those containing new oil. However, the situation was reversed in the sample containing 15% of new oil. The sample containing 5% of new oil had the lowest porosity share among the samples containing engine oil. In the case of these samples, the share of porosity was higher the higher the share of oil in the mixture. Additionally, the inclusion of used engine oil increased the porosity share in the samples.

The reference sample had the highest average pore size value. The samples with the addition of oils yielded significantly lower values. The dependencies for the obtained results were analogous to those obtained for the share of porosity in the samples. In the case of samples with an admixture of vegetable oil, the largest average pore size was characteristic of the sample with an admixture of 10% of new oil, while the smallest was characteristic of the sample with an admixture of 5% of oil after use. For samples containing an engine oil admixture, 15% of old engine oil yielded the highest value, while 5% of new engine oil yielded the smallest value.

### 4.4. Thermal Conductivity

**Figure 15:**
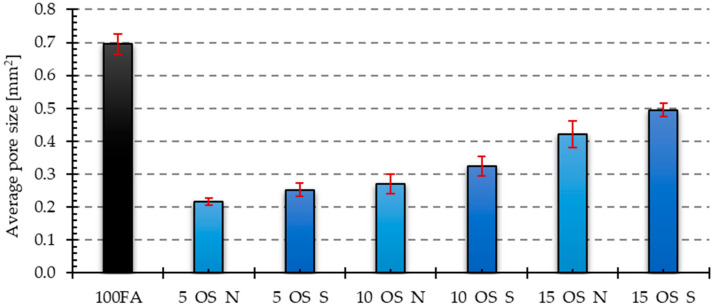
Thermal conductivity of samples with vegetable oil addition in the as-delivered state and after use.

**Figure 16:**
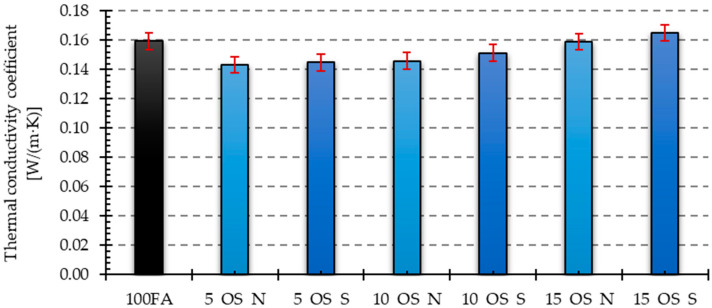
Thermal conductivity of samples with engine oil addition in the as-delivered state and after use.

Thermal conductivity was measured using a lambda meter for the temperature range of 0–20 °C. The results obtained for the measurement of the thermal conductivity coefficient are presented in the form of bar graphs for the variant of samples with the addition of vegetable and engine oil in the delivery state and after use (Figure 15 and Figure 16).

The sample with a 5% oil after use recorded the lowest value of thermal conductivity among samples with vegetable oil additions, nearly 29% lower than the reference sample’s value. Similarly to the density case, we observe that adding oil after use results in lower values compared to adding new oil to the mixture in the same proportions.

A much smaller effect was noted on the value of the thermal conductivity coefficient in samples with the addition of engine oil. The lowest value of the thermal conductivity coefficient was obtained for the sample with the addition of 5% new engine oil, and this value was only about 10% lower compared to the value obtained for the reference sample. As the percentage of oil in the mixture increases, the thermal conductivity coefficient also increases, in line with the density measurement results. However, the sample that added 15% oil after use had a value that was approximately 3.5% higher than the reference sample. When analyzing the effect of the engine oil condition, it can be found that for the same weight shares of oil in the mixture, the density values obtained are higher for the variant that uses used oil.

In the case of samples with the addition of vegetable oil, the lowest value was recorded for the sample with the addition of 5% oil after use, for which this value was almost 29% lower compared to the value obtained for the reference sample. Similarly to the case of density, it can be seen that in the case of using the additive of oil after use, the obtained values are lower than in the case of new oil introduced to the mixture in identical proportions.

Zhang et al. [36], in their work, emphasized the important role of phase composition in determining the functional properties of geopolymer composites. In the studies presented in this paper, it can be observed that the introduction of additives to oils—especially used oils—led to noticeable changes in the geopolymer matrix, as evidenced by increased porosity and changed pore structure.

### 4.5. Strength Properties

**Figure 17:**
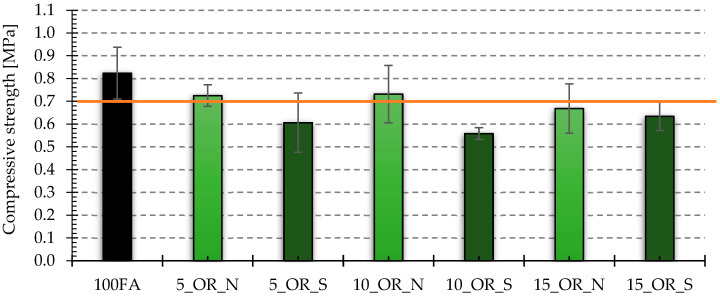
Compressive strength test results for samples with vegetable oil in the as-delivered and after-use conditions. The orange line is a compressive strength threshold of 0.7 MPa typical for infra-lightweight concrete (density below 800 kg/m^3^).

**Figure 18:**
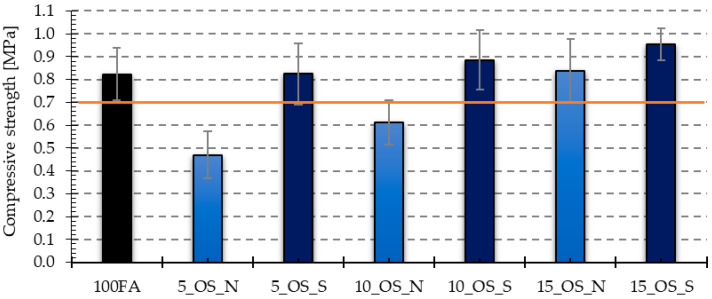
Compressive strength test results for samples with engine oil in the as-delivered and after-use conditions. The orange line is a compressive strength threshold of 0.7 MPa typical for infra-lightweight concrete (density below 800 kg/m^3^).

**Figure 19:**
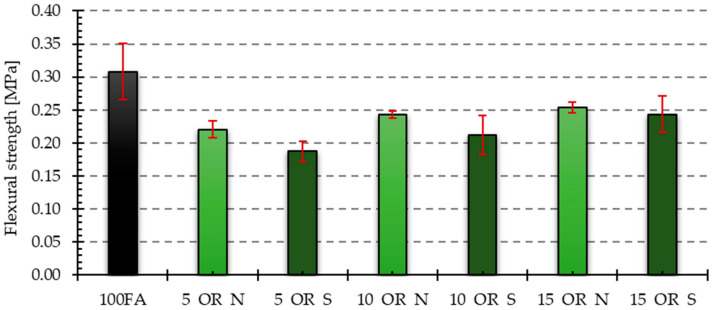
Bending strength test results for samples with vegetable oil in the as-delivered and after-use conditions.

**Figure 20:**
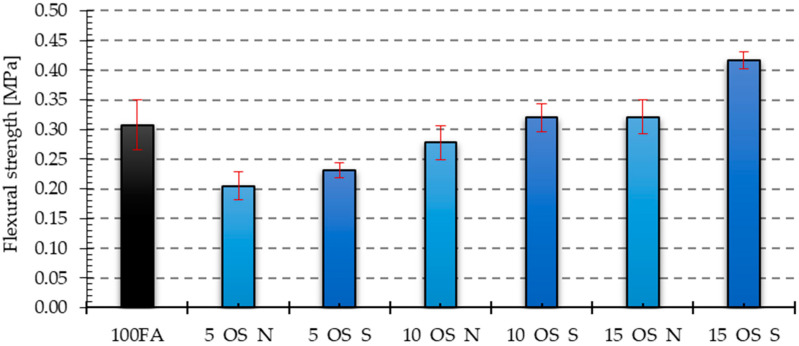
Bending strength test results for samples with engine oil in the as-delivered and after-use conditions.

Figure 17, Figure 18, Figure 19 and Figure 20 show the results of tests that measured the compressive and bending strengths of all the geopolymer samples that were tested. The presented results are average values of measurements carried out on four and eight samples for bending strength and compressive strength, respectively. Based on the obtained results, the standard deviation is determined and plotted as error bars on the graphs. The samples were divided into those that had vegetable oil and engine oil added, both in their as-delivered state and after use.

The average compressive strength value for samples containing vegetable oil showed lower values both during delivery and after use compared to the reference sample, which had a compressive strength of 0.82 MPa. In addition, the condition of the oil was also important, and for samples with the addition of oil after use, lower values were recorded. In the case of samples with the addition of engine oil, only samples with the addition of 5 and 10% of new oil had lower average compressive strength values obtained compared to the reference sample. For the remaining samples, the obtained values were higher, and the highest compressive strength was obtained for the sample with the addition of 15% of old engine oil, and it was almost 16% higher compared to the compressive strength of the reference sample.

Based on the previously discussed results, it can be concluded that the obtained direct compressive strength values are related to the density and pore morphology of the tested samples. The obtained results of the compressive strength measurement for the produced foamed geopolymers can be compared with infra-lightweight concrete, which includes concrete with a density below 800 kg/m^3^ [39] and for which the compressive strength value should be 0.7 MPa [40]. Assuming the value of 0.7 MPa as the cut-off value, it can be seen that the samples with compressive strength below this limit were, in the case of using the vegetable oil additive, samples with a 15% share of new oil and all samples with the addition of this oil after use, while in the case of samples with the addition of engine oil, these were only two types of samples with the addition of 5 and 10% of oil in the delivery state.

Similar relationships, as in the case of compressive strength, were also obtained in the case of bending strength. For samples with the addition of vegetable oil, all of them had lower bending strength compared to the reference sample, for which the bending strength was 4.28 MPa. The lowest bending strength was characteristic of the sample with the addition of 5% of old vegetable oil and its value was almost 39% lower than that of the reference sample. In the case of samples with the addition of engine oil, only the samples with 5% of new and post-use oil and 10% of new oil showed lower bending strength compared to the reference sample. The remaining samples had higher strength, and the highest of them was the sample with the addition of 15% post-use oil, amounting to 5.83 MPa, which is about 136% of the bending strength of the reference sample.

The introduction of vegetable oils into the geopolymer matrix initiates the saponification reaction in an alkaline environment [22]. This initiates the formation of soap compounds that contribute to the development of macropores that can contribute to the reduction in the density and compressive strength of the material while increasing its thermal insulation properties. Used engine oils contain oxidation by-products such as aldehydes, ketones, and other contaminants. These compounds can interfere with the geopolymerization process, causing the formation of larger, irregular pores that adversely affect the material’s mechanical strength.

In the case of the use of new vegetable oils, a tendency to produce more uniform pore structures was demonstrated, which allowed for obtaining higher compressive strength. In this case, it could be concluded that the lack of contaminants in the new oils led to a more predictable interaction with the geopolymer matrix, which resulted in more consistent cross-linking and material integrity. However, this relationship did not hold for the addition of engine oil.

Analyzing the literature reports, in order to improve the strength properties, alternative methods of fly ash modification, such as CO_2_ carbonation, could be considered. This method not only improves the strength of fly ash-based materials but also reduces CO_2_ emissions to the atmosphere. As shown in the studies of Ngo et al. [41], fly ash carbonation increases the initial strength by 40.6%, which allows the use of this material in various engineering processes, such as mining fillings.

### 4.6. Microstructure

**Figure 21:**
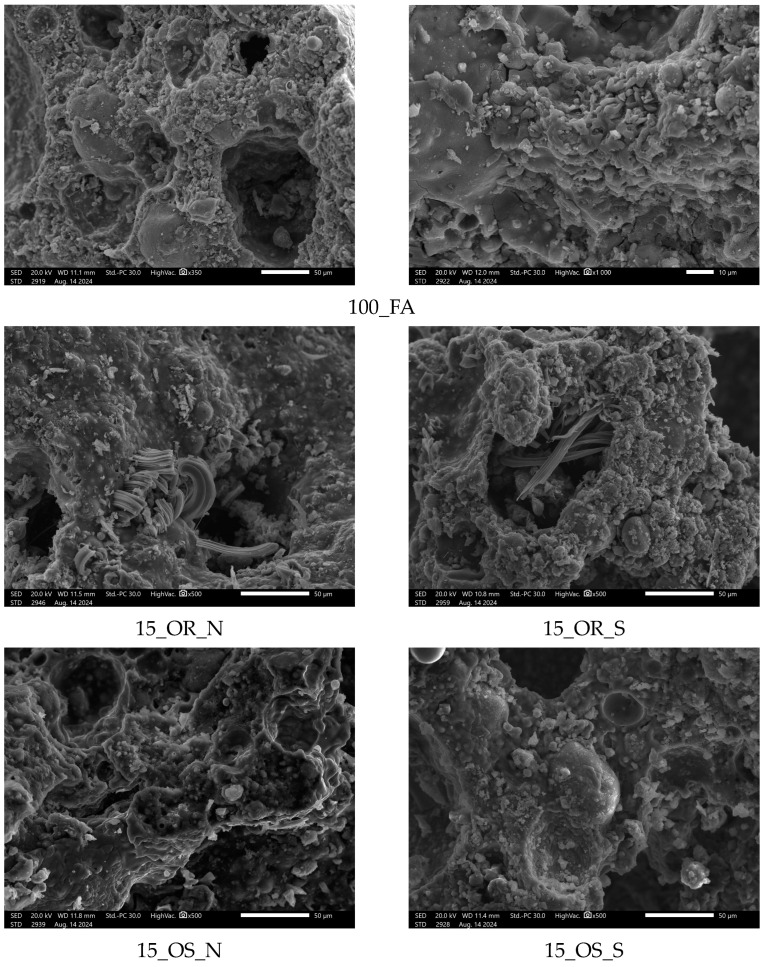
Microstructures of selected samples.

The micrographs presented below (Figure 21) show the structures of the selected geopolymers studied.

The photos show the microstructure of various geopolymer samples, including the reference sample 100_FA and samples with 15% oil added (vegetable and engine), both in the delivery state and after use. In the case of sample 100_FA, the microstructure is characterized by a fairly uniform porosity and a smooth surface, with visible large pores, which is typical for this type of geopolymers.

In the samples with oil added (15_OR_N, 15_OR_S, 15_OS_N, 15_OS_S), a more complex structure can be seen; during microscopic observations, a larger number of pores and channels could be observed compared to the base sample, which indicates a more diverse spatial distribution. The changes in porosity are related to the effect of oils on the cross-linking process of geopolymers. The structure in these samples seems to be more developed and complex, with more fine pores and local protrusions, which may affect the mechanical properties of these materials, such as their strength.

Furthermore, in the samples with the addition of vegetable oil (15_OR_N, 15_OR_S), numerous fiber-like structures are clearly visible, which indicates the presence of salt efflorescence, resulting from chemical reactions between the geopolymer components and the added oil.

### 4.7. Leachability

Table 4 presents the results of the leachability and pH tests of selected samples. The table also includes the permissible leachability limits specified in the Regulation of the Minister of Economy of 16 July 2015 (Journal of Laws 2015, item 1277) [42], applicable in the territory of the Republic of Poland.

Analyzing the obtained results in relation to the permissible leachability limits, it should be stated that due to the significant amount of leached arsenic, selenium, sulfates, dissolved organic carbon and solid dissolved compounds (TDS), waste generated after the production of the tested geopolymer materials should be stored in hazardous waste landfills. Furthermore, when 15% vegetable oil was added to fly ash-based gopolymers, it significantly increased the amount of dissolved organic carbon in the tested samples during leaching, surpassing the limit ranges specified in the Minister’s Regulation. For this reason, the use of vegetable oil for the production of foamed geopolymers based on fly ash should be seriously considered. In order to improve the leachability results, the material used in the work should be subjected to degassing and rinsing in water, as indicated in their work by Łach et al. [43].

Several studies have shown that alkaline-activated geopolymers and binders are much better at effectively immobilizing heavy metals [43,44,45,46] than traditional materials made from Portland cement. Despite the general advantages of geopolymer technology, there are cases where the leaching of some substances, such as chlorides, may exceed the applicable standards, which is why it is indicated that prior treatment of waste is necessary to limit the release of such substances [43,47]. Studies have shown that such pretreatment, especially the washing of waste water, improves the strength of geopolymers compared to materials containing unwashed ash. Additionally, IR spectroscopic analysis has shown that geopolymers containing water-washed ash form more chemically stable structures, isolating heavy metals more effectively [21,43].

Considering the importance of both promoting waste recycling and reducing carbon dioxide emissions, the environmental potential of geopolymers as sustainable building materials is explored. The results of studies incorporating waste oils into geopolymer matrices, which enhance both environmental sustainability and material performance, are also presented in this paper.

The use of recycled waste oils in the production of fly ash-based geopolymers offers significant cost advantages, as waste oils are inexpensive and often readily available. Combined with an energy-efficient geopolymerization process, this approach makes the material economically viable for industrial-scale production. Also, the use of the direct oil addition method simplifies the production process, allowing easy scaling to various industrial applications, especially in the construction sector.

Oil-modified geopolymers exhibit enhanced thermal insulation and lightweight properties, making them ideal candidates for use in the construction industry for thermal insulation panels, lightweight concrete blocks and fire barriers. These materials could also find applications in energy-efficient building systems. In order for the materials discussed in this paper to be suitable for industrial applications, they must meet certain standards of compressive strength and thermal insulation. Therefore, they were compared with infra-lightweight concrete (compressive strength below 0.7 MPa and density below 800 kg/m^3^). Our results suggest that the materials can be optimized to meet such criteria, especially when lower compressive strength is acceptable in exchange for better thermal insulation, and this should be the focus of future research.

Moreover, future research on geopolymers could focus on assessing the suitability of oil-based geopolymers for environmental purposes. Verification of their efficiency in wastewater treatment, for example, through immobilization and adsorption of pollutants, would allow their application to be extended beyond construction to environmental protection technologies. The tunneled porosity and sorption properties of such geopolymers could provide a novel solution in the field of water and wastewater treatment.

## 5. Summary and Conclusions

Geopolymers present an environmentally friendly alternative to traditional building materials, although their use remains limited. This study demonstrates that incorporating vegetable and engine oils, both fresh and used, as additives in fly ash-based foamed geopolymers significantly affects material properties:

- **Density and Pore Morphology**: The addition of oils influences the foaming process, resulting in varied densities and pore shapes. Samples with 5% used vegetable oil had the lowest density (approx. 15% lower than the reference), whereas samples with higher oil content exhibited increased density. The addition of vegetable and engine oils, both in the as-delivered and used state, allowed for a significant reduction in the size of the pores, and their shapes became more spherical compared to the reference sample, which was characterized by the largest pores, the most irregular shapes and their uneven dispersion.
- **Thermal Conductivity**: Samples with uniform porosity, especially those containing 5–10% vegetable oil, achieved a 29% reduction in thermal conductivity, compared to the reference.
- **Strength properties**: The compressive strength of the foamed geopolymers correlates with density and pore structure. Proper oil selection allows the production of geopolymers with strength properties suitable for use as infra-lightweight concrete substitutes.
- **Environmental Concerns**: Significant leaching of arsenic, selenium, and other contaminants suggests the necessity of hazardous waste storage for production residues. Additionally, using vegetable oil may increase dissolved carbon levels, which warrants careful consideration.

In summary, this study demonstrates that the addition of both new and used vegetable and engine oils significantly affects the mechanical and thermal properties of fly ash-based geopolymers. The use of waste oils, in particular, not only enhances the thermal insulation properties but also contributes to sustainable development by recycling industrial waste. These findings suggest that oil-modified geopolymers have significant potential for industrial applications in the construction of lightweight, insulating materials.

Future work should focus on scaling up production processes and tailoring material properties to meet specific industrial standards. Additionally, a cost analysis should be conducted to evaluate the economic feasibility of large-scale production using waste oil additives.

## Figures and Tables

**Table 1:** Sample designation based on the type and amount of introduced additives of vegetable and engine oils, both new and used.

Sample Designation	Weight Share (%)				
	Fly Ash	Vegetable Oil		Engine Oil	
		New	Used	New	Used
100FA	100	-	-	-	-
5_OR_N	95	5	-	-	-
5_OR_S	95	-	5	-	-
10_OR_N	90	10	-	-	-
10_OR_S	90	-	10	-	-
15_OR_N	85	15	-	-	-
15_OR_S	85	-	15	-	-
5_OS_N	95	-	-	5	-
5_OS_S	95	-	-	-	5
10_OS_N	90	-	-	10	-
10_OS_S	90	-	-	-	10
15_OS_N	85	-	-	15	-
15_OS_S	85	-	-	-	15

**Table 2:** Statistical values of fly ash grain size.

	D_10_ (μm)	D_50_ (μm)	D_90_ (μm)	Mean Size of Particles (μm)	Span
Measurement I	2.10	11.10	25.49	13.74	1.895
Measurement II	2.07	11.02	25.53	12.98	
Measurement IIII	2.01	10.78	24.17	13.03	
Average	2.06	10.97	25.06	13.25	
Standard deviation	0.04	0.14	0.63	0.35	0.024

**Table 3:** Oxide composition of fly ash.

Oxide Composition (wt.%)							
SiO_2_	TiO_2_	Fe_2_O_3_	Al_2_O_3_	CaO	MgO	K_2_O	Ball
55.89 ± 0.159	0.785 ± 0.015	4.932 ± 0.02	31.871 ± 0.359	2.82 ± 0.014	0.103 ± 0.006	2.767 ± 0.022	1.632

**Table 4:** The leachability test results for selected samples and the permissible leaching limit values applicable in the Republic of Poland (Journal of Laws of 2015, item 1277).

Sample Designation	Criteria for Admission of Hazardous Waste ^1^	100FA (mg/kg)	15_OR_N (mg/kg)	15_OR_S (mg/kg)	15_OS_N (mg/kg)	15_OS_S (mg/kg)
Arsenic	25	11	17	7.6	18	13
Barium	300	0.36	1.3	0.85	0.095	0.18
Cadmium	5	<0.0020	<0.0020	<0.0020	<0.0020	<0.0020
Total chromium	70	0.89	0.6	0.69	1.4	1.5
Copper	100	0.021	0.15	0.18	0.092	0.12
Mercury	2	<0.010	<0.010	<0.010	<0.010	<0.010
Molybdenum	30	1.9	6.6	5.3	3.1	3.4
Nickel	40	0.013	0.036	0.027	0.028	0.027
Lead	50	0.034	<0.020	0.021	0.031	<0.020
Antimony	5	0.39	0.5	0.29	0.58	0.39
Selenium	7	2.9	2.6	1.9	3	1.9
Zinc	200	0.063	0.18	0.16	0.075	0.1
Chlorides	25,000	520	3100	880	2300	450
Fluorides	500	39	51	41	35	27
Sulfates	50,000	36,000	46,000	27,000	39,000	34,000
Soluble organic carbon	1000	980	14,000	21,000	970	960
TDS dissolved substances	100,000	98,000	56,000	63,000	84,000	65,000
pH of the tested material	-	11.2	11	11.6	12	11.9

^1^ The permissible leaching limits for waste stored in landfills equipped with systems for collecting leachate that is then sent to sewage treatment plants, with the exception of DOC and TDS components, are considered to be met for values higher than those specified in the table.

## Data Availability

Data are contained within the article.
